# Malaria research and its influence on anti-malarial drug policy in Malawi: a case study

**DOI:** 10.1186/s12961-016-0108-1

**Published:** 2016-06-01

**Authors:** Chikondi Mwendera, Christiaan de Jager, Herbert Longwe, Kamija Phiri, Charles Hongoro, Clifford M. Mutero

**Affiliations:** University of Pretoria Centre for Sustainable Malaria Control (UP CSMC), School of Health Systems and Public Health (SHSPH), University of Pretoria, Private Bag X363, Pretoria, 0001 South Africa; Department of Basic Medical Sciences, College of Medicine, University of Malawi, Blantyre, Malawi; School of Public Health and Family Medicine, College of Medicine, University of Malawi, Blantyre, Malawi; Population Health, Health Systems and Innovation, Human Sciences Research Council (HSRC), Pretoria, South Africa; International Centre of Insect Physiology and Ecology, Nairobi, Kenya

**Keywords:** Malaria, Anti-malarial drug policy, Chloroquine, Sulfadoxine-pyrimethamine, Lumefantrine-artemether, Malawi

## Abstract

**Background:**

In 1993, Malawi changed its first-line anti-malarial treatment for uncomplicated malaria from chloroquine to sulfadoxine-pyrimethamine (SP), and in 2007, it changed from SP to lumefantrine-artemether. The change in 1993 raised concerns about whether it had occurred timely and whether it had potentially led to early development of *Plasmodium falciparum* resistance to SP. This case study examined evidence from Malawi in order to assess if the policy changes were justifiable and supported by evidence.

**Methods:**

A systematic review of documents and published evidence between 1984 and 1993, when chloroquine was the first-line drug, and 1994 and 2007, when SP was the first-line drug, was conducted herein. The review was accompanied with key informant interviews.

**Results:**

A total of 1287 publications related to malaria drug policy changes in sub-Saharan Africa were identified. Using the inclusion criteria, four articles from 1984 to 1993 and eight articles from 1994 to 2007 were reviewed. Between 1984 and 1993, three studies reported on chloroquine poor efficacy prompting policy change according to WHO’s recommendation. From 1994 to 2007, four studies conducted in the early years of policy change reported a high SP efficacy of above 80%, retaining it as a first-line drug. Unpublished sentinel site studies between 2005 and 2007 showed a reduced efficacy of SP, influencing policy change to lumefantrine-artemether. The views of key informants indicate that the switch from chloroquine to SP was justified based on local evidence despite unavailability of WHO’s policy recommendations, while the switch to lumefantrine-artemether was uncomplicated as the country was following the recommendations from WHO.

**Conclusion:**

Ample evidence from Malawi influenced and justified the policy changes. Therefore, locally generated evidence is vital for decision making during policy change.

## Background

Research is critical in providing information that can be used for decision making and policy change [1, 2]. For instance, WHO emphasized the importance of evidence when developing policy recommendations on the use of Intermittent Preventive Treatment of malaria in pregnancy with sulfadoxine-pyrimethamine (SP) after reviewing published evidence from various research findings in malaria-endemic regions, including Malawi [3]. On the other hand, experience from many countries has shown how research conducted within the country informs policy [1]. As such, research conducted within a country with valid results is more appropriate to be used to inform policy even though evidence from multi-country studies is more effective for convincing policymakers [1].

However, despite the overwhelming scientific evidence, policy change is not straight forward since it takes into consideration many factors, including the political environment, costs of alternative choices and stakeholders’ views [4, 5]. Choosing the right drug that is efficacious in the treatment of a disease is one step towards policy change, but the change process is often long and tedious, as it involves various stakeholders from both the public and private sectors [5, 6].

The treatment of uncomplicated malaria has, over the years, undergone transitions worldwide, owing to the development of resistance of the *Plasmodium* species to first-line anti-malarial drugs [7]. In a few countries in sub-Saharan Africa (SSA), such as Zambia, Kenya and Tanzania, efficacy data from in vivo studies on chloroquine (CQ) resistance led to policy changes in anti-malarial drug treatment from CQ to SP [1, 8, 9]. However, in most SSA countries, the process for health policymaking has proven to be a complex process [6, 10, 11]. For instance, experience from drug policy change in Kenya, from CQ to SP, revealed difficulties in translating data and the process was complicated, with limited options, unknown adverse effects of replacement therapies, cost, and the limited guidance on factors pertinent to changing the drug policy for malaria [9]. In addition, many of the SSA countries are poor and policy change decisions are highly influenced by their economic budget considerations [12]. This was the case in Sudan, where the decision to change the policy for anti-malaria drug treatment was delayed despite the evidence of drug resistance to CQ [13].

Malawi, as one of the resource-limited countries in SSA, experienced changes in anti-malarial drug policies amid concerns over *P. falciparum* resistance to the first-line anti-malarial drugs and became the first country to change the treatment policy from CQ to SP in 1993 [14] and later to lumefantrine-artemether (LA), an artemisin-based combination therapy (ACT), in 2007 [15]. However, despite the historical changes in first-line anti-malarial treatment regimens, in particular from CQ to SP, many questions were raised as to whether the change had been done too early and whether the new drugs would develop resistance quickly [16]. These concerns were raised as a result of uncertainty surrounding the usage of clear-cut evidence on drug efficacy from within the country or region.

A systematic review and documents review were conducted to examine whether evidence from past research on anti-malarial drug efficacy conducted in Malawi influenced anti-malarial drug policy changes from CQ to SP and SP to ACT, amidst economic, political and health systems challenges. In addition, views from key informants were sought on their experience and general perceptions on the policy changes. Results from this case study provide valuable insights into whether the policy changes were justifiable amidst the challenges and the unforeseen uncertainties with the anti-malarial drug policy change in Malawi.

### Conceptual framework

A case study approach was adopted in order to understand specific issues that were involved in the anti-malaria drug policy changes. This approach was appropriate as it sought to gain an in-depth understanding of the basis for the policy changes in consideration to the concerns raised. A case study attempts to gain an insight into a single occasion on how it occurred through the experiences of those directly involved in the process. Therefore, getting a few answers from the individuals involved in the case enriches the study itself [17].

This case study forms part of the process in understanding the usage of malaria research with the objective of developing a framework that can be used to facilitate the utilization of malaria research evidence for policy development in Malawi. The main purpose of developing this framework was to facilitate adoption of malaria research for policy development, hence maximizing on the limited resources available in the country. It was therefore guided by exploring the institution set up and the barriers and facilitators on the evidence-to-policy process in Malawi.

One of the most important aspects for policy change is the availability of evidence to justify the change. As previously mentioned, the policy changes that occurred in Malawi came with many reservations. Therefore, the basis for the policy decisions needed to be justified. One of the justifications is the availability of evidence. This case study explores whether there was sufficient research evidence to justify Malawi’s policy changes. This study was conceptualized under the philosophy that sufficient and locally generated evidence is required to justify policy change.

This paper focuses on the availability of research evidence; hence, it highlights evidence from efficacy studies on levels of first-line anti-malarial drug regimens and their alternative drugs, which formed the basis for decision making in the policy changes. In addition, the views of key individuals who were directly involved in the policy changes with regards to the policy changes are presented.

## Methods

The study involved three approaches namely, systematic review of published evidence, review of key documents and key informant in-depth interviews.

### Systematic review

A comprehensive literature search was conducted in September 2014. Relevant articles were also searched using the bibliography of all reviewed articles. Combinations of the following specific key words relating to malaria drug efficacy were searched by using the Medical Subject Heading (MeSH) strategy: chloroquine, Fansidar or sulfadoxine-pyrimethamine, Fanasil, pyrimethamine drug combination, lumefantrine-artemether or artemether-lumefantrine combination, and sub-Saharan Africa or Malawi. The search included articles from the periods 1984 to 1993, when CQ was the first-line anti-malarial drug for uncomplicated malaria in Malawi, and from 1994 to 2007, when SP was the first-line drug before being replaced by LA. The following combinations were used during the search: (“key word”[Supplementary Concept]) AND “Malawi”[Mesh] Filters: From 1984/01/01 to 1993/12/31, (“key word”[Supplementary Concept]) AND “sub-Saharan Africa”[Mesh] Filters: From 1984/01/01 to 1993/12/31), “key word”[Mesh] AND “Malawi”[Mesh] AND (“1994/01/01”[PDAT] : “2007/12/31”[PDAT]), “key word”[Mesh] AND “sub-Saharan Africa”[Mesh] AND (“1994/01/01”[PDAT] : “2007/12/31”[PDAT]). The databases searched were Ovid, MEDLINE, PubMed, and Google scholar.

### Selection criteria

Randomized control trials and cohort studies were included on the basis of the following criteria: (1) studies on treatment efficacy for CQ, SP and LA, and (2) studies comparing the efficacies of first-line drugs, i.e. CQ or SP with alternative drugs. Two independent co-authors judged the eligibility of the studies and disagreements were resolved by consensus.

### Analysis approach

Quality assessment of the papers was conducted using the Munn et al. [18] newly developed and tested tool for the critical appraisal of prevalence studies. The purpose was to check whether the research conducted provided tangible evidence for policymaking. This involved examining the methodology used and the findings of the study in comparison to the recommended WHO guidelines to prompt anti-malarial drug policy change.

### Document review

Key documents narrating the process of change such as memos, minutes and reports were sought. In addition, anti-malarial drug policy documents [19–21] were reviewed to examine the extent to which they made reference to the published research and, in this way, establish the link of the study findings with policy and guideline development.

### Key informant interviews

This involved interviewing individuals, such as policymakers and researchers, who were directly involved in the policy changes in order to capture their views on how evidence was utilized and their general opinions on the changes. Hence, a purposive sampling technique was employed in identifying the key informants. In total, 12 individuals were identified and interviewed. There were 10 senior malaria researchers who were involved in the production of evidence used during the policy decisions, of which five were part of the national malaria advisory committee and two were policymakers, including the director for the National Malaria Control Programme. Table 1 highlights the experience, current position and role played by the key informants during the policy changes.

**Table 1:** Details of key informants (KIs) including their roles in the policy changes

KI	Sex	Current position	Experience	Role during policy change
1	Male	Child health and development specialist	9 years current position	Researcher
2	Male	Pharmacologist, College of Medicine (COM)	Over 10 years in malaria research	Researcher
3	Male	Medical epidemiologist – Director of Malaria Alert Center (MAC), COM	10 years current position	Researcher and advisor
4	Male	Senior Scientist, Malawi-Liverpool-Wellcome Trust Clinical Research Programme	More than 40 years in paediatric malaria research	Researcher and advisor
5	Female	Retired Paediatrician and Director of MAC, COM	More than 30 years in clinical and malaria research	Researcher and advisor
6	Male	Paediatrician, Ministry of Health	More than 30 years in clinical and malaria research	Researcher and advisor
7	Male	Paediatrician, Ministry of Health	More than 40 years in clinical and malaria research	Researcher and advisor
8	Male	Clinical Trialist	6 years in current position	Researcher
9	Male	Entomologist, MAC, College of Medicine	Over 10 years current position	Researcher
10	Male	Medical epidemiologist, College of Medicine	More than 15 years in maternal and child health	Researcher
11	Male	Chief of Health Services – Ministry of Health	More than 10 years in clinical and malaria research	Researcher and policymaker
12	Female	Director of the National Malaria Control Programme, Ministry of Health	5 years current position	Policymaker

All the interviews were conducted by the Principle Investigator, who was able to probe and explore in-depth issues based on the conceptual framework of the study. The interviews were conducted in English using a semi-structured interview tool, whose development was guided by the interview schedule for assessing research utilization in policymaking [11].

### Ethics and consent approval

Ethical approval was sought from the Malawi National Health Sciences Research Committee and the University of Pretoria Faculty of Health Sciences Research Ethics Committee during the protocol development. The participants were requested to provide consent approval, to interview and record, before the interviews.

### Themes covered in the in-depth interviews

The interviewees were asked about their perceptions on the policy changes with specific themes covering (1) the availability of evidence for decision making during the policy changes, this verified whether there was enough evidence to form the basis of the decisions that led to the policy changes, (2) the timing of policy changes, this explored if the policy changes were justifiable and made at the right time, and (3) challenges encountered during the policy changes.

### Data management and analysis

The recordings were transcribed and coded based on the themes, the software Nvivo 9 was used to organize the data, while verbatim quotes were used to illustrate concepts and support the conclusions, and in order to bring reality to the situations studied.

Analysis was based on Giorgi’s phenomenological approach, which focuses on the experiences that participants have undergone or through shared life experiences from others that influence their perceptions. This approach documents the findings from the interviewee’s point of view in order to collect the descriptions of their lived world with respect to interpretations in meaning of the phenomena being described [22].

## Results

### Systematic review

**Fig. 1:**
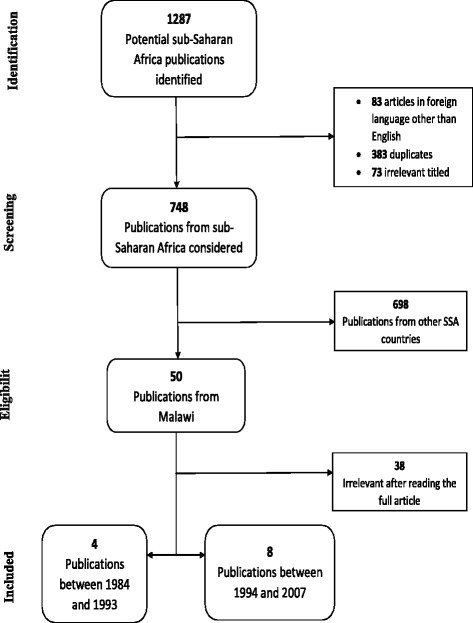
A flow chart of the selection process of publications for inclusion in the review

A total of 1287 relevant publications from SSA were identified using the developed systematic review criteria. After applying the inclusion and exclusion criteria, 12 publications from Malawi remained, with four articles identified from 1984 to 1993 and eight articles from 1994 to 2007 (Fig. 1).

### Publications between 1984 and 1993

The review identified four studies that qualified for analysis (Table 2). Khoromana et al. [23] instituted a study that explored the efficacy of CQ in children under five at two doses of 10 mg/kg and 25 mg/kg. The study was conducted in six sentinel surveillance sites in Malawi across the three regions where malaria transmission occurs throughout the year. The results from these sites were similar. The overall parasitological failure on day 7 of follow-up of CQ was 57%, ranging from 41% to 65%. Despite the poor parasite clearance even at a higher dose, CQ was retained as the first-line drug for the treatment of uncomplicated malaria because of its lower cost and availability compared to alternative therapies.

**Table 2:** Characteristics of malaria publications from Malawi between 1984 and 1993

No.	Publication	Drug(s) under study	Study objective and type	Study population	Protocol used for assessment	Approach	Outcome measured	Results found	Conclusion
1	Khoromana et al. [23]; year of study, 1984	CQ	To assess the appropriate CQ dosage to be used in the Combating Childhood Communicable Diseases program in Malawi	224 children under five presenting at the six outpatient facilities	Modified WHO 7-day in vivo test (1984)	Two CQ dosages of 10 mg/kg and 25 mg/kg were administered	Parasite reduction and clinical response	84% of children given the 10 mg/kg dosage had detectable parasites on day 7, while 57% of 25 mg/kg dosage had a detectable parasite density	Considering the study results and the higher cost and limited availability of alternative therapies, CQ 25 mg/kg therapy was adopted as the primary therapy for malaria
2	Heymann et al. [24]; year of study, 1985	CQ, AQ, SP or Fansidar	To test alternative drugs in children under five	Children under five (39 receiving CQ, 39 at 10 mg/kg AQ, 36 at 25 mg/kg AQ, and 34 at 25 mg/kg SP)	WHO (1984) modified 7-day in vivo test and 21-day follow-up for recrudescence	A comparative trial of AQ in doses of 10 and 25 mg/kg, SP at 25 mg/kg, and CQ at 25 mg/kg	Parasite clearance by day 7; recrudescence at day 21 for AQ 25 mg/kg and SP 25 mg/kg	Parasite clearance of 59% in 25 mg/kg CQ dose, 90% in 10 mg/kg AQ dosage, 97% in 25 mg/kg AQ dosage, and 100% clearance in 25 mg/kg dosage; 34% of recrudescence in the 25 mg/kg AQ group and no recrudescence in the SP group (the results were significant; *P* = 0.01)	The results suggested that, in Malawi, AQ and SP are superior to CQ in producing prompt parasite clearance among young children, and that SP alone is superior to the 4-aminoquinolines in sustaining *P. falciparum* clearance
3	Heymann et al*.* [26]; year of study, 1988	CQ	Experimental study to evaluate the protective efficacy of CQ on *P. falciparum*	334 pregnant women in four antenatal clinics		*P. falciparum* infection rates were measured before and after a 4-week period of CQ prophylaxis	*P. falciparum* parasites in thick smear	48% had *P. falciparum* infection before prophylaxis and 37% had the infection after prophylaxis, making the protective efficacy of CQ at 23%	Research needs to be further conducted to define more cost-effective interventions, including more effective drugs, and health education programmes to improve compliance among pregnant women
4	Bloland et al. [25]; year of study, 1990	CQ and SP	Evaluation of drug efficacy for both short-term parasitological and clinical response to therapy and the long-term implications of the persistent parasitemia	153 children under five attending the outpatient department	Modified WHO in vivo test (1973)	28-days follow-up period on two groups; 124 given CQ and 37 SP	Parasitological resistance	82.3% on parasitological resistance occurred in the CQ group, while 70% in SP group exhibited a parasitological response	Children treated with SP maintained clinical improvement and improved haemoglobin concentration during the follow-up period than those treated with CQ; therefore, CQ was no longer considered as an adequately effective therapy of clinical treatment of malaria in very young children

AQ, amodiaquine; CQ, chloroquine; SP, sulfadoxine-pyrimethamine

Heymann et al. [24] carried out a study that compared the efficacy of CQ to amodiaquine (AQ) and SP in children under five. Parasitological failure on day 7 of follow-up for CQ was at 41%, while AQ had a 97% clearance and SP had a 100% clearance. On the 21-day follow-up period, AQ and SP were further compared in relation to recrudescence, with 34% of recrudescence occurring in the AQ group while none occurred in the SP group. Bloland et al. [25] compared the efficacy of CQ and SP in Kenya and Malawi and results from Malawi showed that there was 82.3% parasitological failure in the 28-day follow-up period in the CQ group, while 70% in the SP group exhibited parasitological response. Bloland et al. [25] concluded that CQ was no longer an effective drug for treating malaria and hence recommended SP as its replacement.

Later, Heymann et al. [26] performed an efficacy study of CQ on parasitaemia during pregnancy. The study found that CQ had a 23% protective efficacy; 37% of the subjects had *P. falciparum* infection despite taking CQ during the study period.

### Publications between 1994 and 2007

The era of 1994 to 2007 saw the use of SP as the first-line drug for the treatment of uncomplicated malaria. The review identified eight studies that qualified for analysis (Table 3). Nwanyanwu et al. [14] examined the efficacy of SP when it had just been adopted after anecdotal and written reports about *P. falciparum*’s resistance to SP. The study found that SP was still very efficacious as it showed parasite clearance in 98.6% of the subjects by day 7 of follow-up. Similarly, Verhoeff et al. [27] conducted a study to assess the efficacy of SP just 2 years after its introduction. SP was found to be efficacious as it had a 90.5% parasitological success clearance rate on day 14 of follow-up. These findings built confidence in the efficacy of SP and removed uncertainties about the drug among clinicians. Nwanyanwu et al. [28] assessed the efficacy of SP 5 years after its widespread use. The study was conducted in seven sites across the country – three with high transmission and four with low transmission. The study found that parasitological resistance to SP (RII and RIII) ranged from 7% to 19%; with one site reaching up to 36%. The level of treatment failure was at 0.9%. It was thus concluded that the efficacy of SP remained at an acceptable level and should therefore be maintained as the first-line drug for treatment of uncomplicated malaria. Takechi et al. [29] assessed the status of anti-malarial drugs in Malawi through an in vivo study for SP only and in vitro study for SP, CQ, mefloquine, quinine and halofantrine. The in vivo results showed that SP was efficacious by clearing the parasites by day 14 of follow-up in 83.1% of the patients, while 13.8% of the patients failed to clear the infections by day 7 (RII/RIII). The in vitro study, however, showed dissimilar results, as 62.1% of the isolates showed resistance to SP, while resistance was only 3.4% in CQ, 3.2% in mefloquine, 5.7% in quinine and 5.9% in halofantrine. Although SP showed significant parasite clearance, the rate of failure had increased from less than 3% found by Nwanyanwu et al*.* [14] to 13.8% by Takechi et al. [29], indicating a deteriorating efficacy of SP.

**Table 3:** Characteristics of malaria publications from Malawi between 1994 and 2007

No.	Publication	Drug(s) under study	Study objective and type	Study population	Protocol used for assessment	Approach/methods	Outcome measured	Results found	Conclusion
1	Nwanyanwu et al. [14]; year of study, 1994	SP	To determine the level of SP efficacy amid numerous anecdotal reports of widespread parasite resistance to SP	145 children under five attending the outpatient clinics	28-day follow-up modified WHO in vivo test (1973)	Half a tablet of SP and half tablet of paracetamol for 3 days	Parasite density	97.9% exhibited parasitological resistance/sensitive pattern, 98.6% had parasite clearance by day 7	These data showed that after 1 year of widespread use of SP in Malawi, *P. falciparum* parasite resistance remained very low contradicting reports of widespread parasite resistance to SP
2	Verhoeff et al. [27]; year of study, 1995	SP	To determine the parasitological and haematological response to SP after being adopted as the first-line drug for treating uncomplicated malaria	84 children under five attending the outpatient clinic with uncomplicated malaria infection	28-day follow-up modified WHO in vivo test (1994)	SP was given according to guidelines of half a tablet to children under 4 years and one tablet to those over 4 years	Parasitological success rate clearance rate and the haematological recovery	90.5% parasitological success rate, while the haematological recovery was not significantly different for parasitological successes or failures	These results showed that, 2 years after the introduction of SP in Malawi for the treatment of uncomplicated *P. falciparum* malaria, the drug combination remained effective in 90.5% of cases
3	Nwanyanwu et al. [28]; study period, 1997–1998	SP	To assess the efficacy of SP 5 years after its widespread use as the first-line drug for uncomplicated malaria	641 children under five attending outpatient clinics in selected hospitals were studied	WHO (1996) 28-day modified in vivo test	Children were treated with the standard malaria treatment guidelines and follow-up examination on days 3, 7 and 14	Parasitological and clinical response	Parasitological resistance (RII and RIII) ranged from 7% to 19%, with one clinic reaching 36%); 0.9% of the patients met the WHO clinical failure by day 7	It was found that, after more than 5 years of widespread use of SP in Malawi, its efficacy remained acceptable for treatment of uncomplicated malaria, and it was therefore, recommended to be retained as first-line treatment
4	Takechi et al. [29]; year of study, 1998	SP, CQ, MF, QN, and HF	To assess the status of antimalarial drug resistance in Malawi	60 children under five attending the outpatient clinic, while in in vitro study, 29 isolates of *P. falciparum* were tested for SP, 29 for CQ, 31 for QN, 29 for HF, and 26 for MF	For in vivo study, WHO (1973) protocol for parasitological follow-up was done at days 3, 7, and 14 after treatment, while an in vitro micro test kit was used to assess susceptibility of *P. falciparum* to the drugs	In vivo efficacy study for SP and in vitro sensitivity study for SP, CQ, MF, QN and HF	Parasite clearance for the in vivo study, inhibition of schizont maturation for in vitro study	In vivo test showed 83.1% RI/S resistance, while in vitro, 62.1% isolates showed resistance to SP, 3.4% in CQ, 3.2% in MF, 5.7% in QN and 5.9% in HF	The results suggested possible recovery of CQ sensitivity after long-term absence of drug pressure, although resistance remained a major problem in malaria control, while in vitro monitoring provides early warning signs of drug efficacy loss, and may detect changing patterns in alternative drug resistance
5	MacArthur et al. [16]; year of study, 1998	SP and MF	A randomised trial to compare the efficacy of SP and MF, a potential successor amid reports of *P. falciparum* resistance to SP	102 children under five attending the outpatient clinic qualified for the study	A modified 14-day WHO (1996) in vivo protocol	40 children were randomized to receive SP 25 mg/kg, and 54 received MF 15 mg/kg	Parasitological response, clinical failure and haematological response	20% combined RII/RIII parasitological failure in SP and 22% in MF; 81.4% had Adequate Clinical Response in SP group and 89.8% in MF group; haemoglobin increase of 1.82 ± 2.29 g/dL in SP and 1.64 ± 1.67 g/dL in MF (*P* = 0.70)	With the decreasing efficacy of SP as the first-line antimalarial drug and the high failure rates of MQ at the tested lower dosage, Malawi should consider assessing the efficacy and feasibility of alternative drugs for treatment of uncomplicated malaria
6	Sulo et al. [30]; study period, 1997–1999	Lapdap and SP	A randomized clinical trial to assess whether Lapdap results in higher retreatment rate for malaria than SP	500 children under five with uncomplicated malaria at the outpatient clinic	WHO (1996) protocol follow-up on days 7 and 28 and thereafter active follow-up was every 28 days with the aim to complete 12 months of follow-up	A group of 222 given Lapdap, another group of 224 given SP	Annual malaria incidence and treatment failure	Mean annual malaria incidence was 2.2 in the Lapdap group and 2.8 in the SP group; 5.4% treatment failure in Lapdap group and 20.5% in the SP group	Despite the rapid elimination of Lapdap, children treated with Lapdap did not have a higher incidence of malaria episodes than those treated with SP; treatment failure was more common with SP
7	Plowe et al. [32]; period of efficacy monitoring, 1998–2002	SP	A prospective open label drug-efficacy study to measure the efficacy of SP in treating falciparum malaria from 1998 to 2002	1377 patients aged 3 months or over presenting at a health centre with uncomplicated malaria	The standard 14 days and 28 days of follow-up	Standard treatment SP doses	Therapeutic efficacy and parasitological resistance	80% of adequate clinical response rate throughout the 5 years and significant decrease in RI parasitological response	Contrary to expectations, SP retained good efficacy after 10 years of use in Malawi and other countries can benefit from interim use of SP while awaiting implementation of combination antimalarial treatments
8	Msyamboza et al. [33]; study period, 2004–2005	SP	To determine the rate of parasitological failure after SP treatment in pregnant women	74 pregnant women presenting with uncomplicated malaria at the clinic	WHO (2002) in vivo protocol	The standard treatment dose was used and a follow-up at days 3, 7 and 14	Parasitological failure	11% parasitological failure	The prevalence of anaemia was high at first antenatal visit and the rate of parasitological failure had increased from 5% in 1996 to 11% in 2004; but the low prevalence of malaria in the population could indirectly indicate acceptable SP drug sensitivity

CQ, chloroquine; HF, halofantrine; QN, quinine; Lapdap, Chlorproguanil-dapsone; MF, mefloquine; SP, sulfadoxine-pyrimethamine

MacArthur et al. [16] conducted a clinical trial to compare the efficacy of SP and mefloquine as an alternative drug after surveillance data had indicated *P. falciparum*’s resistance to SP. The study showed poor efficacy on day 14 of follow-up in both SP and mefloquine, as a combined parasite failure of RII and RIII were 20% and 22%, respectively. The MacArthur et al. [16] study was one of the early studies to report on the deteriorating efficacy of SP. Therefore, the authors recommended that other alternative drugs should be considered and tested. Sulo et al. [30] conducted a year-long study in Kenya and Malawi to measure the annual incidence of malaria in two groups that were treated with either SP or chlorproguanal-dapsone (Lapdap) as an alternative therapy. The 7-day follow-up period results from Malawi showed that the mean annual malaria incidence was 2.8 compared to 2.2 in the groups treated with SP and Lapdap, respectively. There was a 5.4% treatment failure in the Lapdap group compared to 20.1% in the SP group. However, Lapdap was later found to have major negative side effects and was withdrawn [31]. In other related studies, Plowe et al. [32] published their findings from an open-label drug-efficacy test for SP that was conducted from 1998 to 2002. The authors established that SP had maintained a good efficacy rate during the 14-day follow-up period from the time it had been adopted, with a clinical response rate of 80% or higher. Msyamboza et al. [33] conducted their study to assess the efficacy of SP in pregnant women in a rural clinic with high malaria transmission. They found that parasitological failure was at 11%. Msyamboza et al.’s [33] findings showed that resistance patterns in pregnant women followed those observed in children under five and the level of SP efficacy was still at an acceptable level.

#### Document review

Availability of records posed a major challenge, in particular minutes and memos were not available for the assessment. Therefore, document review was based on a report outlining the policy change from CQ to SP [34], the 2005 Malawi anti-malarial drug efficacy study [35], and the anti-malaria drug policy guidelines [19–21].

### The change from CQ to SP

In 1984, Malawi established its basic structure for the malaria control program, whose duty was the development of the national malaria control policy to guide interventions aimed at addressing the malaria burden in the country. The policy outlined a 5 year plan including the guidelines for malaria treatment. It was recognized that evidence was critical in the development of this policy. Therefore, several studies were conducted between 1984 and 1989 that provided evidence for the malaria treatment policy development.

In this regard, an understanding of the dynamics of malaria as a disease in children was required and hence the National Malaria Research Project instituted operational research with the aim of assessing the impact of malaria in children and to improve treatment strategies. This study found that the overall infant mortality rate in Mangochi district was at 163 per 1000 live births, with a neonatal mortality rate of 49 per 1000 births and a post-neonatal mortality rate of 111 per 1000 births. However, no specific cause of death was identified in the neonatal period, yet in the post-natal period malaria-related symptoms were identified to be associated with the deaths. It was also revealed that 70% of the deaths occurred within 7 days of the onset of illness. The study further found that almost two thirds of the deaths occurred at home with 53% and 70% of deaths in neonates and post-neonates, respectively.

This study played a major role in defining the impact of malaria. The findings showed that a high infant mortality rate occurred in rural Malawi and provided an estimate of deaths attributed to malaria with recognition that most deaths occurred in the community than in health facilities. These findings were significant in the formulation of the 1990 National Plan for Malaria Control in which strategies for implementation were improved by emphasizing the prompt identification and treatment of malaria in children at community level. For policy implementation, it ensured the availability of drugs at all levels of healthcare, training of community volunteers and health workers in effective case management, and the need for rolling out health education in the communities to inform caregivers on recognizing malaria-related symptoms and seeking effective malaria treatment.

Concerns were also raised about the emerging of CQ resistance and the need of assessment of the malaria policy, which led to the recognition of a systematic approach in evaluating the malaria control policies. One of the strategies was the identification of six sentinel sites in Malawi for surveillance of malaria focusing on parasitological and clinical response of anti-malarial drugs. This involved in vivo studies testing the efficacy of CQ in single doses of 10 mg/kg and 25 mg/kg body weight [23].

In vivo drug efficacy studies for alternative drugs to replace CQ were also conducted involving two doses of AQ at 10 mg/kg and 25 mg/kg, and SP at 25 mg/kg [24].

For purposes of understanding the clinical response, a study was undertaken to compare the clinical response of children to CQ and SP. The study looked at three clinical indicators that included the presence of at least 75% of children with *P. falciparum* infection and correlated with parasite density, history of fever during the preceding 48 hours, and history of altered activity level during the preceding 48 hours, and axillary temperature of greater that 37.2 °C. The findings showed that, on the second day, CQ had a rapid effect in addressing symptoms consistent with rapid schizonticidal activity and antipyretic effect. However, on the seventh and fourteenth days the clinical failure rates for CQ were not significantly higher than for SP.

The findings from these studies were utilized in developing the malaria therapy policy in 1985, which resulted in discontinuing the routine treatment of children under five and the country adopted the presumptive treatment of fever with CQ at a dose of 25 mg/kg. The studies also influenced the retention of CQ as the first-line drug in treating uncomplicated malaria despite parasite resistance, since it demonstrated a positive clinical response by the seventh day of treatment, and its wide availability and general safety in its use. It was recommended from the findings that AQ and SP become therapies of choice after CQ treatment failure in children under five.

The increased concerns of CQ resistance led to implementation of a re-evaluation of in vivo studies to assess the efficacy of CQ, SP and AQ at the dosages of 25 mg/kg. The findings showed the deterioration of CQ efficacy while AQ showed poorer response on day 7 compared to SP indicating parasite resistance. These studies were, however, not considered sufficient to warrant policy change although a routine drug assessment policy was put in place.

The national malaria control committee evaluated its initial 5 year plan from 1984 to 1989 to feed into its next strategic plan from 1989 to 1993. One of the major findings from this evaluation was the maintained rapid increase of malaria-related morbidity and mortality as the hospitalizations of children under five increased by 43% and malaria case fatality rose by 30%. Therefore, the operation research agenda focused on supporting studies that could provide evidence in refining the malaria control policy. The main area of focus was the assessment of alternative drugs that could replace CQ for treatment of uncomplicated malaria. In addition to this, the studies assessed the clinical, haematological and parasitological drug response. Based on these studies conducted in Malawi, WHO adopted the qualification of an anti-malarial drug as efficacious for use in treating uncomplicated malaria in children under five if it was able to adequately alleviate the symptoms of the disease, clear the parasites and allow a tolerable parasite-free interval for haematological recovery [25].

Bloland et al. [25] conducted and published the Karonga and Mangochi follow-up studies that compared the parasitological and clinical responses of CQ and SP. The publication has also been reviewed in the systematic review. As a direct result of these studies, the treatment guidelines of the first-line drug for uncomplicated malaria changed from CQ to SP in 1992. The policy change was fully implemented in 1993, when adequate stocks of SP were procured. SP was also advantageous since it was easy to administer as it was tasteless and required a single dose leading to increased compliance.

### Sentinel surveillance report for the change of SP to LA

In January 2005, the National Malaria Control Programme convened a meeting with its supporting committees – the Malaria Advisory Committee and the National Malaria Technical Committee. They discussed, among other things, the need to change the country’s first-line anti-malaria drug policy after considering that efficacy studies had shown SP 14-day clinical treatment failure of above 15% that recommended by WHO as the cut-off point, prompting policy change. Although WHO had recommended ACTs [36] to be the best option for first-line anti-malarial drugs, there was a need to generate local evidence in order to make an informed choice on the optional ACTs available. Therefore, open label randomized efficacy trials targeting children under five in three sentinel sites during the malaria season commenced in April 2005. The objective of the study was to assess four drug combinations (AQ plus artesunate (AQ-Art), AQ plus SP (AQ-SP), chlorproguanil-dapsone plus artesunate (CD-Art), and LA). In addition, the efficacy of SP was also conducted for comparison purposes to the new drugs.

The results showed poor efficacy of SP, as it had only 32% of adequate clinical and parasitological response on day 28, while the other drugs showed over 90%, specifically 100% in AQ-Art, 95% in AQ-SP, 94% CD-Art, and 93% in LA. Thus, all the combination drugs were similar in superiority to SP. These findings provided the local evidence for the Malawi government to choose the most appropriate combination drug for treating uncomplicated malaria. Finally, LA was the suitable choice in replacing SP.

### Policy and guideline documents

The anti-malarial treatment policies and guidelines were reviewed to assess how they made reference to research evidence that informed their development. A major challenge with these documents is that they did not have a formal reference section that could be assessed as to what specific study was used in their development. However, the documents clearly mention the in vivo studies conducted in the sentinel sites as the major sources of local evidence that influenced policy changes. The in vivo studies in the sentinel sites that led to the change of CQ to SP were published and included in the systematic review [25], while the unpublished 2005 in vivo efficacy studies provided the evidence for policy change of SP to LA [35].

#### Findings from the key informant interviews

Key informants provided their experiences and views towards the policy changes and how evidence was critical in driving the changes. As previously indicated, the main concern for the historical change from CQ to SP was the uncertainty on the sustainability of SP’s high efficacy on *P. falciparum*. Hence, the question was whether the change was justifiable at that time. Therefore, views of key informants were sought regarding the change.

### Availability of evidence for justification of policy changes

Clinicians and other medical personnel in health facilities observed that CQ was no longer effective in the treatment of malaria. As narrated by one of the researchers, who was also a clinician:

“*In the mid-1980s, around 1985, clinicians across the country started observing that people treated with chloroquine were coming back complaining that they do not feel better*”.

### Timing of policy changes

The several reports from the clinicians raised concerns about using CQ in treating malaria, which prompted the government of Malawi through the Ministry of Health to conduct efficacy studies that would provide empirical evidence to substantiate these reports. The results of these studies revealed that indeed CQ was not working and there was need for change [25]. This was acknowledged by one researcher, who is also a paediatrician:

“*It was timely, the evidence was clear that chloroquine wasn’t working at the time, I’m a child specialist so I actually see the effects or the complications, if the drug is not working, the children do not get well, some even die against severe forms of malaria, so seeing that we decided to make a change, it was quite obvious we needed the change*”*.*

Another researcher also recounted the need for local evidence to base the policy decision on:

“*And then of course there a was also need for the research to be done to back up the actual change and again for Malawi that change came about because of drug efficacy studies that were carried out over the years which then meant that it was easy for the policy makers to certainly say hey its indeed high time to change because this drug is obviously not working*”.

### Challenges during policy changes

The main challenge for this change however, was that there were no clear WHO guidelines for policy changes, i.e. on what recommended drugs to replace CQ, hence there was limited support from WHO, as stipulated by one researcher:

“*The first challenge was the fact that there was no buy-in from WHO.... so WHO was saying who is going to pay if we are not supporting it…who is going to pay for this?*”

This situation led to Malawi making a strong case of changing the policy since WHO was concerned with early development of parasite resistance to SP. However, based on the data generated locally, increased morbidity and mortality were a major concern for Malawi. Hence, further studies were initiated to gather strong evidence. This was confirmed by one researcher who, on what led to them conducting efficacy studies, said:

“*That then prompted government to consult Center for Disease Control to help out with investigations on what was happening, why are several patients treated coming back complaining of the same signs and symptoms?*”

Therefore, evidence was generated and presented to the policy decision makers for their next step. It further showed that the policy change from CQ to SP needed to be based on tangible, structured evidence. Hence, for this purpose, the studies provided the evidence required.

From this case study it can be observed that records were well documented and published regarding the change from CQ to SP and those studies were published during this time, including in the early years of change to SP compared to the change of SP to LA. As highlighted from one researcher:

“*Well at this stage from SP to LA they were following the WHO recommendations so I suppose people didn’t worry too much about it, you know, getting track with what actually was happening but with the earlier period when we had to change from chloroquine to SP this was new but the advantage we had was that we had locally generated data that could not be refuted*”.

The WHO plays a vital role in driving policy issues in the world. It will provide guidelines that countries adapt. Recommendations from WHO will easily influence decisions for policymakers as they deem that these recommendations are tangible. WHO recommended that the first-line anti-malarial drugs be replaced by ACT [36]; therefore, in 2005, Malawi had to carry out efficacy studies that included the assessment of SP in comparison to ACTs for possible replacement. Hence, the guidelines were very critical in changing the drug policy from SP to LA as countries, including Malawi, were following these recommendations, but the change from CQ to SP had to rely on strong locally generated evidence to convince policymakers to make the switch since such guidelines did not exist at that time.

## Discussion

Research has been proven to provide vital evidence for decision making and, more critically, for policy development [2]. Research is critical in resource-limited countries such as Malawi in order to maximize the usage of resources. Owing to the constant mutation of the malaria parasite (*Plasmodium sp*.), there has been a major challenge in malaria prevention and control [37], prompting changes in anti-malarial drug regimens across the years in Malawi and other malaria-endemic countries [38]. This case study explored the availability and usage of research evidence that formed the basis for decision making in the policy changes. This was done through a systematic review examining published research evidence on malaria drug efficacy studies conducted between 1984 and 1993, and 1994 and 2007, examination of documents in the form of reports and policy guidelines to assess their reference to evidence, and in-depth interviews with key informants directly involved in the generation of evidence during the policy changes with the objective of soliciting their general views and perceptions towards the policy changes.

It was found out that there was enough scientific evidence from research conducted from 1984 to 1993 on CQ resistance to support a change in anti-malarial drug policy from CQ to SP in Malawi. Secondly, although the evidence did not come from peer reviewed journals, studies from sentinel sites conducted between 2005 and 2007 showed a reduced efficacy of SP within Malawi, influencing a change in first-line anti-malarial treatment from SP to LA. Views of key informants indicate that the policy changes were timely enough, although the change from CQ to SP could have occurred earlier. These results, therefore, support the proposition that evidence from drug efficacy studies within Malawi influenced the changes in policy on anti-malarial drug treatment.

Malawi was the first country in SSA to change its first-line anti-malarial drug from CQ to SP [14] and, in 2007, it changed its policy again from SP to LA [21]. Amid the economic, political and other challenges within the country, concerns were raised during the policy change from CQ to SP as to whether the change had occurred too early and the change’s implications for the quick development of resistance to SP [16]. Studies conducted between 1984 and 1993 provided ample evidence about the poor efficacy of CQ and the superiority of SP as its replacement. The objectives of some of the reviewed studies strongly indicate that the studies were conducted for purposes of policy formulation [23–25, 39]. The study by Khoromana et al*.* [23] was instituted with the sole purpose of guiding malaria treatment drug policy in Malawi. However, despite its findings of low efficacy of CQ in Malawian children, the dosage of 25 mg/kg was adopted in the treatment of uncomplicated malaria as the first-line drug. Similar studies with such objectives to drive policy have also been conducted and published in other countries [40–42]. A case study on Malawi by WHO about the development of anti-malarial drug policy in the period of 1984 and 1993 highlighted the findings by Khoromanana et al*.* [23] and Bloland et al. [25] as having provided the important information critical for policy and guideline changes [43], and these findings were also acknowledged by Nwanyanwu et al*.* [14] to have been used for policy development. These studies were conducted in different geographical regions and in areas of high and low transmission during both rainy and dry seasons. The treatment failure observed from these studies exceeded the 25% recommended by WHO as the cut-off point [43], in this way prompting anti-malarial drug policy change. The studies also presented the findings of alternative drugs studied [24, 25] and have shown evidence that SP was more practical as a replacement for CQ in this period.

Following the change of the anti-malarial first-line drug in 1993 to SP, unconfirmed reports of its poor efficacy subsequently led to five efficacy evaluation studies being undertaken [14, 27–29, 32]. The findings from these studies showed a maintained high acceptable level of efficacy of SP and influenced the policy decision to retain it as the first-line anti-malarial drug for treatment of uncomplicated malaria in Malawi. However, from the publications assessed between 1994 and 2007, the efficacy of SP was still at a level for it not to warrant policy change. Since no publications were found after Plowe et al.’s [32] study, conducted between 1998 and 2002, it would be difficult to ascertain when SP started losing its efficacy to levels prompting its removal as a first-line drug for malaria treatment. However, in 2005, the Malawi government, through the National Malaria Control Programme, carried out unpublished efficacy studies in the sentinel sites in children under five. The programme tested the efficacies of four combination drug candidates: AQ-SP, AQ-Art, CD-Art and LA, while SP was also assessed to provide up-to-date data on its efficacy for comparison with the new drug candidates. SP showed deterioration, as it had only 32% of adequate clinical and parasitological response on day 28, while the other drugs showed over 90% of adequate clinical and parasitological response.

Policy development is intricate and not entirely determined by research evidence [44]. In order to establish the impact of research on policy and practice, reports [34] and the Malawi Ministry of Health anti-malarial drug policy documents [19–21] were reviewed to check for any references made to research. These documents did not have a formal reference section. However, the guidelines clearly referred to regular in vivo studies, as recommended by WHO [37] and conducted in sentinel sites in varied geographical regions of the country, to have been a major factor influencing policy development, especially the change from SP to LA.

Generally, WHO develops and updates protocols that guide anti-malarial drug efficacy studies [45]. This standardization is necessary in order for those involved in research to produce comparable and viable results to help guide policymaking. However, even when not limited to these protocols, utilization of randomized control trials with blinding is essential in order to reduce bias as much as possible [45]. In this study, only two studies explicitly indicated to have used randomization [16, 30], while three studies [16, 23, 30] explained how they arrived at particular sample size using statistical methods. Nevertheless, evidence from the various malaria studies from Malawi showed some agreement in outcome of their findings despite differences in their methodology.

Experiences and views from key informants indicate that Malawi required ample evidence to change its policy from CQ to SP when there were no WHO guidelines to recommend the change. WHO concerns were based on the fact that changing to SP would lead to parasite resistance to SP much earlier, which would also be a concern to neighbouring countries due to border crossing that occur and hence lead to a regional problem. However, Malawi demonstrated that the change was eminent and continual monitoring of SP showed that no parasite resistance developed to levels of concern for policy change until in 2007, when SP eventually was replaced by LA based on WHO recommendations.

As for later changes to ACTs, WHO updated its guidelines for countries to follow when switching their first-line anti-malarial to ACTs [36]. In this regard, many countries made the changes earlier than Malawi, such as Zambia, which became the first African country to change from CQ to LA in 2002 [46]. Kenya switched from SP to LA in 2004 [47] and, by June 2006, 39 African countries had switched to the WHO recommended ACTs [46]. Malawi only made its switch in 2007 as it required gathering thorough local evidence from the sentinel sites. As already highlighted, the change from SP to LA was smooth since it was done following WHO recommendations.

## Conclusions

Substantial malaria drug efficacy studies were conducted in Malawi, which provided tangible evidence for policy decision making. The change from CQ to SP was systematic; at a time when there were no clear WHO guidelines for changing a drug that loses it efficacy, the team from Malawi observed that CQ was no longer efficacious and hence carried out studies that provided strong evidence to justify the change, while unpublished sentinel surveillance studies provided evidence for policy change from SP to LA [21]. Consequently, there was justifiable evidence from efficacy studies conducted within Malawi that were used for timely policy changes.

Based on the findings, strong locally generated evidence is crucial for policy decision making. In addition, the study recommends proper record keeping and that policy documents and guidelines should be formally referenced to allow tracking of evidence used for their development. An example of such referenced malaria guidelines exists in the case of Kenya [48]. In addition, sentinel surveillance findings should be published so that they undergo peer review and become readily available to a wider community. Research and monitoring of drug efficacy should continue to be conducted according to the recommendations of the WHO protocols and methodologies in order to ensure quality of the research results.

## Abbreviations

ACT, artemisin-based combination therapy; AQ-Art, amodiaquine plus artesunate; AQ-SP, amodiaquine plus sulfadoxine-pyremethamine; CQ, chloroquine; CD-Art, chlorproguanil-dapsone plus artesunate; LA, lumefantrine-artemether; Lapdap, Chlorproguanal-dapsone; SP, Sulfadoxine-pyrimethamine; SSA, sub-Saharan Africa
